# Supplementary material for: Causal Relationship between Aging and Anorexia Nervosa: A White-Matter-Microstructure-Mediated Mendelian Randomization Analysis
Source: Biomedicines. 2024 Aug 16;12(8):1874. doi: 10.3390/biomedicines12081874 (PMC11351342; doi:10.3390/biomedicines12081874)
Supplement: Supplementary file 1 [file biomedicines-12-01874-s001.zip › Supplementary Figure/Figure S4/Singe SNP plot of MR analysis for TL to ubm-b-2105.pdf]

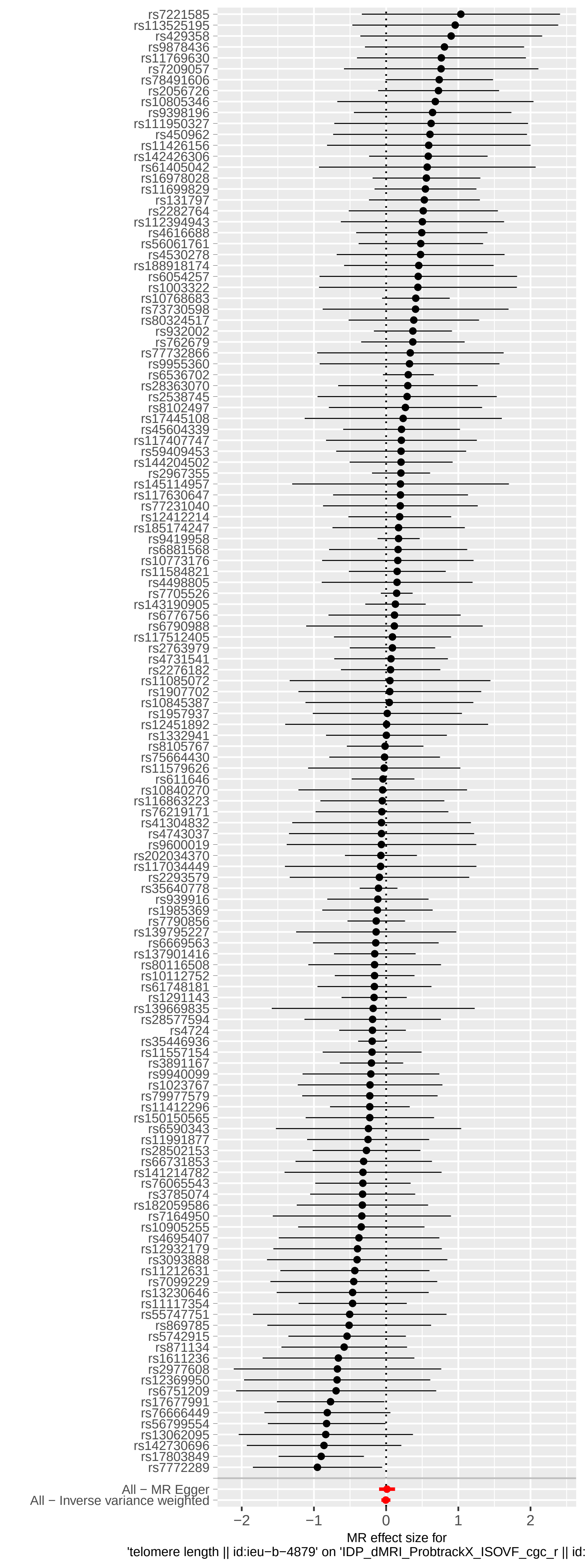
rs7221585
rs113525195
rs429358
rs9878436
rs11769630
rs7209057
rs78491606
rs2056726
rs10805346
rs9398196
rs111950327
rs450962
rs11426156
rs142426306
rs61405042
rs16978028
rs11699829
rs131797
rs2282764
rs112394943
rs4616688
rs56061761
rs4530278
rs188918174
rs6054257
rs1003322
rs10768683
rs73730598
rs80324517
rs932002
rs762679
rs77732866
rs9955360
rs6536702
rs28363070
rs2538745
rs8102497
rs17445108
rs45604339
rs117407747
rs59409453
rs144204502
rs2967355
rs145114957
rs117630647
rs77231040
rs12412214
rs185174247
rs9419958
rs6881568
rs10773176
rs11584821
rs4498805
rs7705526
rs143190905
rs6776756
rs6790988
rs117512405
rs2763979
rs4731541
rs2276182
rs11085072
rs1907702
rs10845387
rs1957937
rs12451892
rs1332941
rs8105767
rs75664430
rs11579626
rs611646
rs10840270
rs116863223
rs76219171
rs41304832
rs4743037
rs9600019
rs202034370
rs117034449
rs2293579
rs35640778
rs939916
rs1985369
rs7790856
rs139795227
rs6669563
rs137901416
rs80116508
rs10112752
rs61748181
rs1291143
rs139669835
rs28577594
rs4724
rs35446936
rs11557154
rs3891167
rs9940099
rs1023767
rs79977579
rs11412296
rs150150565
rs6590343
rs11991877
rs28502153
rs66731853
rs141214782
rs76065543
rs3785074
rs182059586
rs7164950
rs10905255
rs4695407
rs12932179
rs3093888
rs11212631
rs7099229
rs13230646
rs11117354
rs55747751
rs869785
rs5742915
rs871134
rs1611236
rs2977608
rs12369950
rs6751209
rs17677991
rs76666449
rs56799554
rs13062095
rs142730696
rs17803849
rs7772289
All − MR Egger
All − Inverse variance weighted
−2
−1
0
1
2
MR effect size for
'telomere length || id:ieu−b−4879' on 'IDP_dMRI_ProbtrackX_ISOVF_cgc_r || id:
